# Effect of Low Doses (5-40 cGy) of Gamma-irradiation on Lifespan and Stress-related Genes Expression Profile in *Drosophila melanogaster*

**DOI:** 10.1371/journal.pone.0133840

**Published:** 2015-08-06

**Authors:** Svetlana Zhikrevetskaya, Darya Peregudova, Anton Danilov, Ekaterina Plyusnina, George Krasnov, Alexey Dmitriev, Anna Kudryavtseva, Mikhail Shaposhnikov, Alexey Moskalev

**Affiliations:** 1 Laboratory of Post-Genomic Research, Engelhardt Institute of Molecular Biology, Russian Academy of Sciences, Moscow, Russia; 2 Laboratory of molecular radiobiology and gerontology, Institute of Biology, Komi Science Center of RAS, Syktyvkar, Russia; 3 Laboratory of genetics of aging and longevity, Moscow Institute of Physics and Technology, Dolgoprudny, Russia; 4 Department of ecology, Syktyvkar State University, Syktyvkar, Russia; ENEA, ITALY

## Abstract

Studying of the effects of low doses of γ-irradiation is a crucial issue in different areas of interest, from environmental safety and industrial monitoring to aerospace and medicine. The goal of this work is to identify changes of lifespan and expression stress-sensitive genes in *Drosophila melanogaster*, exposed to low doses of γ-irradiation (5 – 40 cGy) on the imaginal stage of development. Although some changes in life extensity in males were identified (the effect of hormesis after the exposure to 5, 10 and 40 cGy) as well as in females (the effect of hormesis after the exposure to 5 and 40 cGy), they were not caused by the organism “physiological” changes. This means that the observed changes in life expectancy are not related to the changes of organism physiological functions after the exposure to low doses of ionizing radiation. The identified changes in gene expression are not dose-dependent, there is not any proportionality between dose and its impact on expression. These results reflect nonlinear effects of low dose radiation and sex-specific radio-resistance of the postmitotic cell state of *Drosophila melanogaster *imago.

## Introduction

Throughout the history of living things, the natural background radiation of the Earth and cosmic rays have been one of the key environmental factors that have affected the rate of evolutionary processes [1, 2]. As a result of nuclear weapons testing, nuclear accidents and the activities of the nuclear fuel cycle, large areas were contaminated with artificial radionuclides [3–5]. Furthermore, additional sources of irradiation are present in medical procedures, air travel and certain manufacturing [6–9]. Thus, the problem of biological effects of low doses of ionizing radiation is becoming increasingly important.

Although there are many common mechanisms of response of organism and cell to irradiation and other stresses (thermal, oxidative etc.) [10], their principal difference is a significant role of DNA damage on the biological effects of ionizing radiation [11, 12]. However, these differences are attributed mostly to high dose rates. In the case of low dose radiation, direct effects of irradiation such as clustered DNA damage and DNA double strand breaks are minimal, whereas indirect DNA damages caused by the induction of reactive oxygen species become the primary result [11, 13]. In high doses, adverse effects accumulate in the tissues in a deterministic manner that depends linearly on the dose, but in low doses the effects are stochastic, non-linear on the dose, and depend mainly on the efficiency of the stress response’s protective mechanisms [14]. Therefore, low doses of radiation can be regarded as moderate stress, which is known to induce hormesis [15]. Indeed, in our previous work [14, 16], and in the work of other authors [17] it has been revealed, that relatively low dose exposure (20–75 cGy) of fruit flies on immature preimaginal stages in some cases has long-term effects that lead to an increased life span and resistance to other stresses, such as hyperthermia [18, 19]. It is known that preimaginal stages of *Drosophila* have comparable radiosensitivity to mammals [20]. At the same time, adult individuals, due to the postmitotic state of most tissues, are about 100 times more radioresistant [21]. In their recent work, Antosh et al. revealed that irradiation of *Drosophila* individuals in the imago stage in doses from 0.1 to 400 Gy causes a statistically significant effect on lifespan and gene expression only if the dose is higher than 100 Gy [22]. At the same time, in our recent work on comparing the effects of irradiation in the adult *Drosophila* male and female at the 20 cGy dose rate, we observed some differentially expressed genes [23].

Therefore, the goal of this work was to identify changes of lifespan and expression of several previously identified low dose radiation-induced genes in *Drosophila melanogaster*, exposed to low doses of γ-irradiation (5–40 cGy) at the imaginal stage of development.

## Materials and Methods

### Experimental design

In our experiments, we used laboratory wild-type (*Canton-S*) males and females. The line was obtained from the collection at the Bloomington *Drosophila* Stock Center at Indiana University (Bloomington, USA).

The control- and experimental flies were maintained at T 25±0.5°C and a 12 hour light regime on a sugar-yeast medium containing 7 g of agar, 30 g of sugar, 8 g of dry yeast, 30 g of semolina, 4 ml of propionic acid, and 1 liter of water. Males and females were kept separately at densities of 30 flies of the same sex and age per 120 mL vials.

For analyzes of the expression profiles, the flies in the imago stage of development were used for each control- and experimental variant. For each variant, 3 biological replicates were pooled. Experimental flies were exposed to gamma-irradiation from ^226^Ra source with the dose rate of 36 mGy/h. The source had metal casing (aluminum filter) impervious to alpha particles, so the spectrum of ionizing radiation had been exposed to gamma irradiation. The exposure time was 1 h 23 min, 2 h 47 min, 5 h 34 min and 11 h 8 min, and the absorbed dose was 5, 10, 20 and 40 cGy, respectively. The control flies were maintained in the same conditions excluding irradiation factor. The flies in the control- and experimental groups were fixed by liquid nitrogen after a specific time following irradiation: immediately after the radiation impact, after 6, 24, 48 and 72 hours and stored in a freezer at -86°C.

The lifespan replicates and the gene expression samples were in one pool, from which the gene expression samples were extracted at fixed time points (0, 6, 12, 48, 72 hours after the exposure).

### Lifespan analysis

For the analysis of the lifespan alterations, 150–170 individuals (males and females were kept separately) were used. Flies were transferred to a fresh medium two times a week. Dead flies were counted daily. For each experimental variant 3 biological replicates were pooled. Two control groups (one–for 5 and 10 cGy, another–for 20 and 40 cGy) for males as well as for females were used, due to the large exposure time difference (1 h 23 min and 2 h 47 min–for 5 and 10 cGy; 5 h 34 min and 11 h 8 min–for 20 and 40 cGy respectively). These replicates were merged, since flies were kept in the same conditions and the similar effects in the same variants were observed.

Survival functions were estimated using the Kaplan–Meier procedure and plotted as survival curves [24]. Median lifespan and the age of 90% mortality were calculated. The statistical analysis of survival data was conducted using nonparametric methods. Comparison of survival functions was done using the modified Kolmogorov–Smirnov test [25]. The statistical significance of differences between the mean lifespans for the experimental and control variants was determined using the Gehan–Breslow–Wilcoxon test [26]. To test the statistical significance of differences in maximum lifespan (age of 90% mortality), the Wang–Allison test was used [27]. Results of the log rank test are presented in the S1 Table.

It is well known that the Gompertz function is applicable for describing *Drosophila* lifespan alterations [28], so we approximate all survival curves with Gompertz equation: µ(*x*) = exp(α*x*) R_0_ [29]. We calculated parameters α and of the Gompertz equation, coefficients of determination that characterize the quality of the Gompertz function approximation [30] and the mortality rate doubling time (MRDT) [30]. Maximum likelihood method was used to evaluate the significance of differences in the intensity of mortality [31]. It's well known that there is a Strehler-Mildvan correlation between α and R_0_ parameters of the Gompertz equation [32]: [32]: ln(R_0_) = γ-βα (α and R_0_ – parameters of Gompertz equation, γ and β –regression parameters).

The Kaplan-Meier curves were plotted using STATISTICA, version 6.1 (StatSoft Inc, USA). Calculation of lifespan parameters and their statistical analysis were performed in the R software environment for statistical computing and graphics (http://www.r-project.org/). WinModest Version 1.0.2. [31] was used to calculate the parameters of the intensity of mortality.

### RNA isolation and cDNA synthesis

Total RNA was isolated from homogenized samples (five flies from every sample) by QIAzol Lysis Reagent (Qiagen, Netherlands) and further isopropanol precipitation. The RNA concentration was determined using a NanoDrop® ND-1000 spectrophotometer (NanoDrop Technologies Inc., USA). The A260/A280 ratio of the RNA samples was 1.8–2.0. The integrity of the isolated RNA (RNA integrity number, RIN) was determined using the Bioanalyzer Agilent 2100 (Agilent Technologies, USA). Only the samples with an RIN value not less than 8.0 were used. Single-strand cDNA was synthesized using 1 μg of total RNA pretreated with DNase I (Fermentas, Lithuania), hexanucleotide primers, and M-MuLV reverse transcriptase (Fermentas, Lithuania) by the following scheme: 10 min at 25°C, 60 min at 42°C, 10 min at 50°C, and 10 min at 70°C.

### qPCR

Real-time PCR was carried out on the 7500 Real-Time PCR System (Applied Biosystems, USA) by using modified short 6-carboxyfluorescein (FAM)-labeled probes from the Universal Probe Library (UPL, Roche, Switzerland). Pairs of primers were selected for every gene with the estimation of probability of primer dimers and heterodimers using OligoAnalyzer (http://eu.idtdna.com/calc/analyzer). The primer sequences are listed in the S2 Table. Each reaction was run 3 times with 10 μL mix, containing PCR-buffer, dNTPs in concentration 250 nM, primers– 300 nM, UPL, ROX, DNA polymerase 1 unit and cDNA diluted 17.5 times. The threshold cycle Ct was determined (7500 Software v2.0.5, Applied Biosystems, USA). The amplification efficiency values were calculated as described earlier [33]. The primers and probes proved to be specific by electrophoresis using Bioanalyzer Agilent 2100 (Agilent Technologies, USA); the size of amplification products were as expected.

### Statistical analysis of qPCR data

The first step of the analysis of qPCR data is the evaluation of the stability of reference genes by four methods ΔCT [34], BestKeeper [35], Normfinder [36], Genorm [37]. The stability of all genes was analyzed relative to each other so the average rating of all genes was obtained by using all four methods. This rating showed the stability of all genes relative to each other in the certain experimental conditions. Only genes with high stability ratings were used as reference genes for expression normalization. The expression of four reference genes *Actin*, *RpL32*, *EF1alpha*, *betaTub* [38] was analyzed. Analysis of expression stability revealed that genes *Actin*, *betaTub* are very variable in this experiment. So only genes *RpL32*, *EF1alpha* were used as reference for expression normalization.

Ct values obtained for each gene in each sample were normalized to the reference gene Ct values for the calculation of the relative gene expression according to the formula:

$\frac{E_{i}^{-Ct_{ij}}}{\sqrt[{n}]{E_{r1j}^{-Ct_{r1j}}*\ldots *E_{rnj}^{-Ct_{rnj}}}}$, where R_ij_ – relative gene expression of i gene in j sample, E_i_, E_r1j_, E_rnj_−efficiency of reaction for gene and reference gene respectively, Ct_ij_, Ct_r1j_, Ct_rnj_−threshold cycle of gene and reference gene respectively. All efficiencies were more than 90%. The expression change compared with control was log_2_FC (Fold Change), where FC = R_iexp_/R_icontrol_ for each biological replicates, then mean log_2_FC was calculated for all biological replicates. All calculations were performed using statistical computing programming language R (version 2.15.1). At least 2-fold mRNA level changes were considered as significant because of reference genes mRNA level variability.

## Results and Discussion

### Lifespan alterations in *Drosophila melanogaster* wild-type *Canton-S* individuals after the exposure to low doses of γ-irradiation

**Fig 1:**
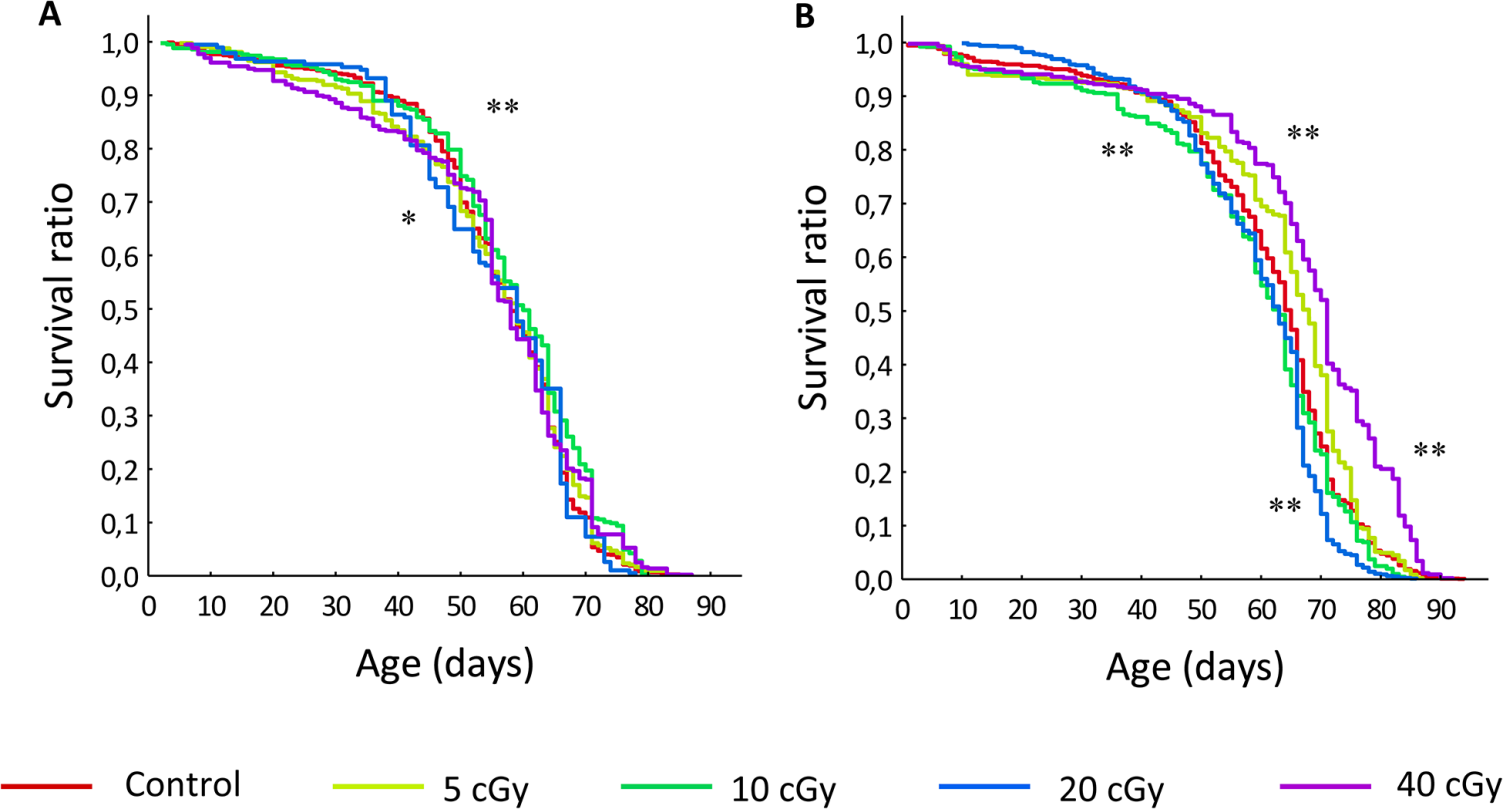
Influence of low doses of γ-irradiation on the lifespan of *Drosophila melanogaster*, wild-type line *Canton-S*. A–males, B–females, *—p<0.05, **—p<0.01, (Kolmogorov-Smirnov test).

In *Drosophila melanogaster* wild-type *Canton-S* males, after exposure to low doses of ionizing radiation, we have observed the effect of hormesis: after the influence of γ-irradiation at a dose of 10 cGy, median lifespan increased by 3.4% (p<0.01, Gehan-Breslow-Wilcoxon test), the maximum lifespan increased by 4.2% (p<0.01, Wang-Allison test), exposure to γ-irradiation at doses of 5 and 40 cGy caused the extension of MRDT by 11.4 and 22.5% (p <0.01 maximum likelihood method), respectively (Table 1, Fig 1A).

In *Drosophila melanogaster* wild-type *Canton-S* females, after exposure to γ-irradiation at doses of 5 and 40 cGy, an increase of median lifespan was observed (by 4.5 (p <0.05, Gehan-Breslow-Wilcoxon test) and 7.6% (p <0.01, Gehan-Breslow-Wilcoxon test) respectively). The impact of radiation at doses of 10 and 20 cGy leads to a decrease in this index by 4.5% (in both cases) (p <0.01, Gehan-Breslow-Wilcoxon test). The maximum lifespan increased by 6.3% after the influence of irradiation at a dose of 40 cGy and decreased after the impact at doses of 10 and 20 cGy by 3.8 and 10.1% (p <0.01, Wang-Allison test). The impact of irradiation at a dose of 20 cGy has revealed itself in decreased MRDT by 19% (p <0.01, maximum likelihood method). According to the above results, we can conclude that hormesis appears in *Drosophila melanogaster* females during the exposition doses of 5 and 40 cGy, and the opposite effect of hyperradiosensivity is demonstrated after irradiation treatment at doses of 10 cGy and 20 cGy (Table 1, Fig 1B).

**Table 1:** Alterations of the lifespan parameters in *Drosophila melanogaster* after exposure to low doses of ionizing radiation.

Sex	Dose	М (day)	ΔM (%)	90% (day)	Δ90%	MRDT (day)	Δ MRDT (%)	α (day^−1^)	R_0_ (day^−1^)	R^2^	N
♂	Control	58	-	71	-	7.52	-	0.092	0.00031	0.805	1044
	5 cGy	59	1.7	71	0	8.38	11.4 (*)	0.083 (*)	0.0005 (*)	0.718	423
	10 cGy	60	3.4 (**)	74	4.2 (**)	7.88	4.8	0.088	0.00032	0.703	426
	20 cGy	59	1.7	70	-1.4	7.35	-2.3	0.094	0.00029	0.743	391
	40 cGy	58	0	71	0	9.21	22.5 (**)	0.075 (**)	0.00071 (**)	0.563	438
**♀**	Control	66	-	79	-	8.64	-	0.08	0.00032	0.77	1017
	5 cGy	69	4.5 (*)	78	-1.3	7.87	-8.9	0.088	0.00019	0.57	381
	10 cGy	63	-4.5 (**)	76	-3.8 (**)	9.06	4.9	0.076	0.00051 (*)	0.63	318
	20 cGy	63	-4.5 (**)	71	-10.1 (**)	7	-19 (**)	0.099 (**)	0.00016 (**)	0.82	457
	40 cGy	71	7.6 (**)	84	6.3 (**)	8.04	-2.8	0.082	0.00018	0.64	438

Table 1 legend: M–median lifespan, 90%–age of death of 90% of the sample (maximum lifespan), MRDT–mortality rate doubling time, ΔM, Δ90% and ΔMRDT–differences with the control for M, 90% and MRDT, α and R_0_ – parameters α and of Gompertz equation, R^2^ – determination coefficient of Gompertz approximation, N–number of individuals in the sample.

*—p<0.05

**—p<0.01, (Wang–Allison test for Δ90%; Gehan–Breslow–Wilcoxon test for ΔM; maximum likelihood method for α and ΔMRDT).

**Fig 2:**
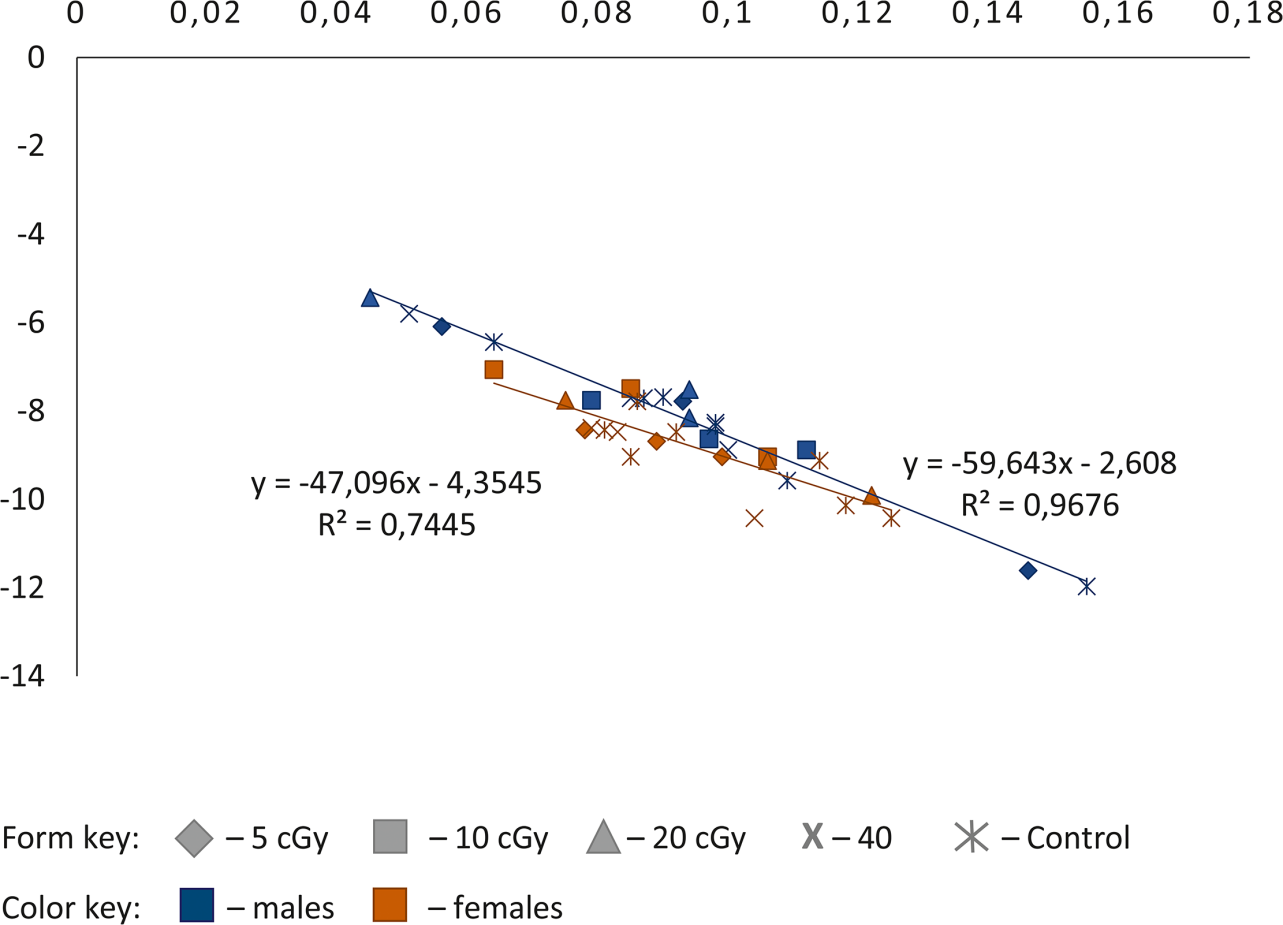
Strehler-Mildvan correlation between the parameters of the Gompertz function in *Drosophila melanogaster* wild-type *Canton-S* individuals exposed to low doses of ionizing radiation.

Fig 2 demonstrates the presence of the Strehler-Mildvan correlation between the parameters α and R_0_ of the Gompertz equation in *Drosophila melanogaster* wild-type line *Canton-S* males and females after the studied exposure doses. Each point on this parametric plane corresponds to the specific survival curve (three replicates per each exposure dose for male as well as for female). Correlation coefficients are equal to—0.98 (р < 0.0001) and—0.93 (р < 0.0001) in males and females respectively. It is known that the link between the parameters of the Gompertz function is equivalent to the presence of the intersection point of the survival curves. Moreover, the abscissa of this point is equal to the regression parameter β of the Strehler-Mildvan correlation equation, that is, the meaning of "typical life expectancy of the population" can be attributed to the value of this parameter [39]. In Fig 2, it is well shown that parameters of the Gompertz equation are approximated by the regression line, which is usual for “normal” physiological conditions [40]. In addition, the α and R_0_ of the Gompertz equation for all groups in males as well as in females do not significantly diverge from the regression line, thus, we can conclude that there are no differences in the "typical life expectancy of the population" between treated and control flies.

We have thus demonstrated the presence of hormesis in *Drosophila melanogaster* wild-type strain *Canton-S* male and female animals after exposure to γ-irradiation at doses of 5 and 40 cGy (according to various criteria). Females have also revealed the effect of hyperradiosensivity after irradiation doses of 10 and 20 cGy. However, it should be noted that because of calculation of the Strehler-Mildvan correlation, it was demonstrated that there are not deviations from the normal organism's physiological functions in treated male and female *Drosophila melanogaster* relative to the control.

Any change in lifespan relates to complex interactions of genetic and physiological factors [41, 42]. It is known that the effect of ionizing radiation in low doses can deviate in the direction of increasing negative consequences (hyperradiosensivity) [43] as well as in the direction of reducing negative consequences (radiation hormesis) [44]. Speaking about the possible mechanisms of radiation-induced changes, we should note that the effects of low doses of ionizing radiation affect the development of the organism, the immune response, lead to a change in the metabolism of proteins, amino acids, lipids, fatty acids, and hormones, alter energy metabolism, lead to tumor necrosis factors induction, cause changes in the cell cycle, in the processes of cell proliferation and differentiation, cause DNA damage, apoptosis, proteolytic degradation, autophagy and oxidative stress [17, 45–48].

For this reason, we investigated the time- and dose-response dependence of the alterations in differential expression of 29 genes involved in the cell stress response, DNA repair, apoptosis, antioxidant protection, and detoxification of xenobiotics using qPCR method.

### Gene expression analysis after low dose radiation exposure

In this work, the dynamics of changes in the expression of stress sensitive genes (Table 2) in response to irradiation by low doses of 5–40 cGy in the *Drosophila melanogaster* wild-type strain *Canton-S* were analyzed.

**Table 2:** The genes selected for expression analysis in the samples of *Drosophila melanogaster* wild-type strain *Canton-S* 72 hours after radiation exposure in doses from 5 cGy to 40 cGy.

Gene	Function	Reference
*Hus1-like*	DNA-damage-induced checkpoint response, activation of an S-phase checkpoint, oocyte DNA organization	[49]
*foxo*	Insulin signaling, resistance against oxidativestress	[50]
*spn-B*	RAD52 DNA repair pathway, double-strand DNA break (DSB) repair, meiotic checkpoint activation	[51, 52]
*p53*	G1 growth arrest, induction of apoptosis, radiation-induced apoptosis	[53, 54]
*mei-41*	Cell-cycle control, post-replication repair	[55, 56]
*DJNK*	Immune response activated by bacterial infection, wound healing, morphogenetic movement during embryogenesis	[57–59]
*tefu*	Spontaneous apoptosis suppression, female fertility, protection from telomere fusion, activation of checkpoint signaling in response to DNA double-stranded breaks induced by low-dose ionizing radiation	[60–62]
*Clk*	Master transcriptional regulator of the circadian clock	[63]
*PCNA*	Control of eukaryotic DNA replication by increasing the polymerase's processability	[64, 65]
*hpo*	Hippo/SWH (Sav/Wts/Hpo) signaling pathway, organ size control, tumor suppression, inhibition of transcriptional complex activity, regulation of Th/DIAP1 apoptosis inhibitor	[66, 67]
*Sod*	Radical detoxification	[68]
*Brca2*	Double-strand break repair by meiotic and mitotic homologous recombination	[69]
*mei-9*	Meiosis recombination events, Holliday junctions within recombination intermediates, repair of mismatches within meiotic heteroduplex DNA, nucleotide excision repair	[70, 71]
*RAD54*	Mitotic DNA repair, meiotic recombination, recombinational DNA repair pathway	[72]
*mus309*	DNA replication, DNA repair, exhibition of a magnesium-dependent ATP-dependent DNA-helicase activity	[73, 74]
*wrinkled*	Apoptosis activation	[75]
*Cyp6a20*	Monooxygenase, oxidoreductase, electron carrier activity, heme binding, iron ion binding, takes part in aggressive behavior and defense response to Gram-negative bacterium	[76–78]
*CG13323*	Unknown function	http://www.uniprot.org
*GstE3*	Glutathione transferase activity, response to oxidative stress, resistance to insecticides	[79–81]
*CG18180*	Serine-type endopeptidase activity, proteolysis with a possible role in immune function	[82–85]
*Keap1*	Actin binding, defends organisms against the detrimental effects of oxidative stress	[86, 87]
*CG42751*	Unknown function	http://www.uniprot.org
*CG6295*	Hydrolase, lipid metabolic process	[88, 89]
*CG6675*	Hydrolase, lipid metabolic process	http://www.uniprot.org
*Fer3*	Transcription factor that binds to the E-box and functions as inhibitor of transcription. DNA binding requires dimerization with an E protein. Inhibits transcription activation by ASCL1/MASH1 by sequestering E proteins	[90, 91]
*CG9360*	Oxidoreductase activity	http://www.uniprot.org
*Cyp4e2*	Metabolism of insect hormones	[92]
*Hsp70Aa*	Recognition of sequences of hydrophobic amino acid residues, transmembrane transport of proteins, cell protection from thermal or oxidative stress, disposal of damaged or defective proteins, apoptosis inhibition	[93–97]
*per*	Period length of circadian and ultradian rhythms, eclosion behavior, male courtship song, circadian transcriptional loop	[98]

The genes *CG13323*, *GstE3*, *CG18180*, *Keap1*, *CG42751*, *CG6295*, *CG6675*, *Fer3*, *CG9360*, *Cyp4e2*, *Hsp70Aa*, *Cyp6a20*, *per* were included in this analysis because previously in our laboratory differential expression of these genes in response to different stress factors including radiation was identified [23]. Other genes, including *Hus1-like*, *foxo*, *spn-B*, *p53*, *mei-41*, *tefu*, *PCNA*, *hpo*, *DJNK*, *Sod*, *Brca2*, *mei-9*, *RAD54*, *mus309*, whose expression were analyzed, are very important in response to stress impact. The regulation of circadian rhythm [99] and apoptosis [100] are also known to be changed by genotoxic stress, and, therefore, the genes *Clk* and *wrinkled* were included in this analysis.

**Fig 3:**
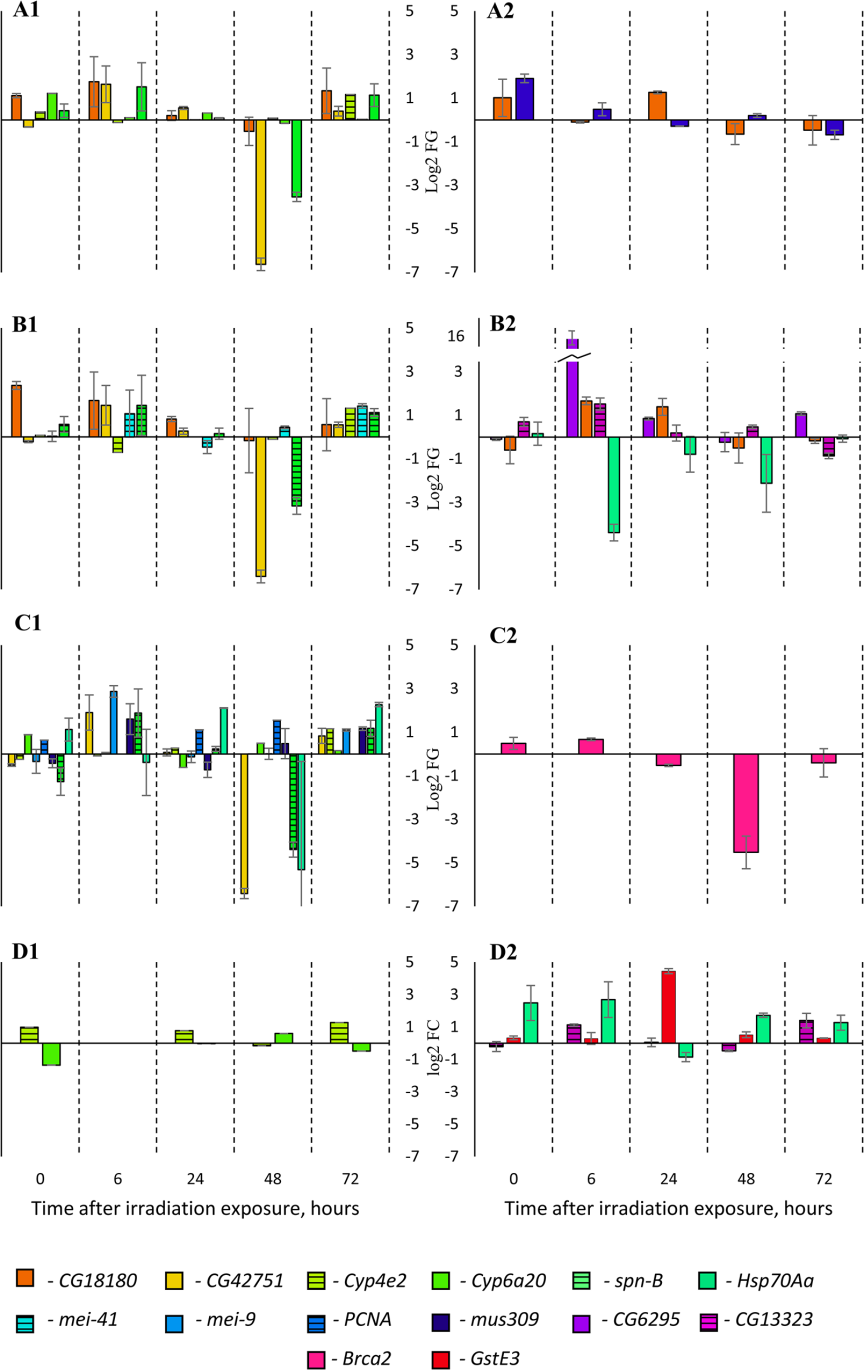
The differentially expressed genes in *Drosophila melanogaster* males and females after the radiation exposure. A– 5 cGy, B– 10 cGy, C– 20 cGy, D– 40 cGy, 1 –males, 2 –females. Only gene changes with Log_2_FC > 1 and p-value < 0.05 during at least one time range are presented.

Data about up- and down expression and p-value obtained for each gene in each sample are shown in Table 3 and raw qPCR data is presented in S1 File. The values were considered as statistically significant if appropriate p-value was less than 0.05. Ct values from qPCR for three biological replicates after the radiation exposure are performed in the S3 Table. The data of the relative expression, log2FC, the mean and the standard deviation are performed on the graphics in S1 File. These graphics show that for some genes under a certain radiation dose and at a certain time after the impact, the standard deviation is very high (more than 5% of mean value) or log2FC is less than 1 (FC is less than 2-fold change in this case). Such genes were identified as non-differentially expressed at this experimental point. In this way, the set of differentially expressed genes for every irradiation dose was obtained (Fig 3). The highest FC effects were performed 48 hours after the impact, but there are very few statistically significant values at this point. Under the radiation impact of a 40 cGy dose, the data are comparable with the biological variability in most cases.

**Table 3:** Analysis of the gene expression by the qPCR in the samples of *Drosophila melanogaster* wild-type strain *Canton-S* 72 hours after radiation exposure in doses from 5 cGy to 40 cGy (Female/male).

Irradiation dose, cGy	5					10				
Analysis time, hours after exposure	0	6	24	48	72	0	6	24	48	72
CG6295	n/n	n/n	-/-	-/n	n/n	n/n	+ */n	n/n	n/n*	+/n
CG18180	n/+ *	n/+	+ */n	n/n	n/+	n/+ *	+ */+	+ */n*	n/n	n/-
CG42751	n*/n	n*/+	n/n*	n/- *	n*/n*	n/n	n/+	n/n	n/- *	n/n
Clk	n*/n	n*/n	n/n	n/n	n/n	n*/n	n/n	n*/n*	n/n	n/n
Cyp4e2	n/n*	n*/n*	n/n*	n*/n*	n*/+ *	n*/n*	n/n*	n*/n	n*/n*	n*/+ *
Cyp6a20	n/+ *	n/n*	n/n*	n*/n*	n/n	n/- *	n/n*	n/n	n/n*	n/+
Fer3	-/-	+/-	n/n	n/n	+/+	n/-	n*/-	n/n	n/n	+/+
foxo	-/n	n/n	n/n	n/n	n/n	n/n	n/n	n*/n	n*/n	n/n*
GstE3	n/n	n/n	+/n	n*/n	n/n	n*/n	n/n	+/n	n*/n	n/n
hpo	n/n	n*/n	n/n	n/n	n/n	n/n	n/n	n*/n*	n*/n	n/n
Hsp70Aa	n*/n	n/+	-/n	n/-	n/n	n/+	- */-	n/n	-/-	n/n
Hus1-like	n/n*	n/n	n/n	n/n	n/n	n/n	n/n	n*/n*	n*/n	n/n*
DJNK	n/n	n/n	n/n	n/n	n/n*	n/n	n*/n	n/n	n*/n	n/+
Keap1	n/n	n/n	-/n	+/n	n/n	n/n	-/n	n/n	+/n	+/n
mei-9	n/n	n/n	n/n	n/n	n/n	n/n	n/n	n/n*	n*/n	n/n
mei-41	n/n	n/n	-/n	n/n	n/+	n/n	-/+	n/n*	n/n	n/+ *
PCNA	n/n*	n/n	n/n	n/+	n/n	n/n	n/n	n/n*	+/+	n/n
mus 309	+ */n	n/n	n/n	n/n	n/n	n/n	n/n	n/-	n/n	n/n
p53	n/n	n/n	-/-	-/-	n/n	n/n	n/n	-/-	-/n	n/n
per	n/n	n/n*	n/n	+/n	n*/n*	n/n	-/n*	n/n*	n/n*	n/n
RAD54	n/n	n/n	n*/n	n/n	n/n	n*/n	n*/n	n/-	n*/n	n/n
Sod	n/n	n/n*	n/n	n/n	n/n	n/n	+/n	n/n*	n/n	n/n
spn-B	n/n	n/+	n/n	+/- *	n/+	n/n*	n/+	n/n	+/- *	n/+
tefu	n/n	n/n	-/n	n/n	n/n	n/n	n*/n	-/n*	n/n	n/n
wrinkled	n/n	n*/n*	n/+	n/+	n/+	n*/-	n/-	n/+	n/+	n/n
CG13323	n*/n	n/n	n*/n	n*/n	+/n	n/n	+ */n	n/n	n/n	n*/n
Brca2	n/n	n*/n	n/n	n*/n	n/+	n/n	n*/n	n*/n	n/n	n*/+
CG6675	n/n	n/n	n/n	n/n	n/n	n*/n	n*/n	n/n	n/n	n/n
CG9360	n/n	n/n	n/n	n/n	n/n	n/n	n/n	n/n	n/n	n/n
Irradiation dose, cGy	20					40				
Analysis time, hours after exposure	0	6	24	48	72	0	6	24	48	72
CG6295	n*/n	n*/n	-/-	n/n*	n/n	+/n	+/-	n/-	+/-	n/+
CG18180	n/n	n/n*	n/n	n/n	n/-	n/n	n/+	+/n	n/n	n/+
CG42751	n/n	n/+	n/n	n/- *	n/n	n*/n	n/+	n/n	n*/-	n/+
Clk	n/n	n/+	n*/n	n/n	n/n	n*/-	n*/n*	+/n	n/n	n/n
Cyp4e2	n*/n*	n*/n*	n*/n*	n*/n*	n*/+ *	n*/n*	n*/n	n*/n*	n*/n*	n*/+ *
Cyp6a20	n/+ *	n/n*	n/n*	n/n*	n/n*	n/- *	+/n	n*/n*	-/n*	n/n*
Fer3	n*/-	n/n	+/n	n/+	n*/+	+/n	n*/-	n/n	n/n	n*/n
foxo	n/n*	n/n	n*/n	n*/n	n*/n	n/n	n/+	n*/n*	n*/n	n/+
GstE3	n/n	n/n	n*/n	n/n	n/n	n*/n	n/+	+ */n	n*/n	n/+
hpo	n/n	n/n	n*/n	n*/n	n/n	n/n	n/n*	n/n	n*/n	n/n
Hsp70Aa	n/+ *	n/n	-/+ *	n*/-	+/+ *	+/+	+/-	n*/-	+ */-	+/n
Hus1-like	n*/n	n/n*	n/n	n*/n	n/n	n/n	n/+	n/n	n*/n	n/n
DJNK	n/n*	n/n	n*/n	n*/n	n/n*	n/n	n/+	n*/n	n*/n	n/n*
Keap1	n/n*	n/n*	n/n	n*/n	+/n	n/n	n*/n	n/n	n*/n	n/n
mei-9	n/n	n/+ *	n/n	n*/n	n/+ *	n/n	n/n*	n/n	n*/n	n/+
mei-41	n/n	n/n	n/n	n/n	n/n	n/n	n/n	-/n	n/n	n/+
PCNA	n/n	n/n	n/+ *	n*/+ *	n/n*	n/n	n/n	n/+	n*/n*	n/n*
mus 309	n/n	n/+	n/n	n/n	n/+ *	n/n	n/n	n/n	n/n	n/+
p53	n/-	n/n	-/-	n*/-	n/n	n/n	n/n	n*/n	n/-	-/+
per	n/n	n/n	n/n	n/n*	n/n	n/n	+/n*	n/n	n/n*	-/+
RAD54	n*/-	n*/n	n*/n	n*/n	n*/n	n/n	+/n	n/-	n*/-	n/n
Sod	n/n	n/n	n/n	n/n*	n/n	n/n	-/n	n/n	-/n	n/+
spn-B	n/-	n/+	n/n*	n*/- *	n/n*	n/n	n/n	n/n	n*/-	n/n
tefu	n/n	n/n*	-/n	n/n	n/n	n/n	n/n	n/n	n/n	n/n
wrinkled	n*/n	n/n	n*/+	n/+	n/n	n*/-	+/-	n*/n	-/-	n/+
CG13323	n/n	n*/n	n/n	n*/n	n*/n	n/n	+ */n	n/n	n/n	+ */n
Brca2	n/n	n/n	n*/n	-*/-	n/+	n/n	n*/n*	n*/n	- */-	n/n
CG6675	n*/n	n/n	n*/n	n/+	n/n*	n/n	n/+	n/n	n/n	n/+
CG9360	n/n	n/n	n/n	n/n	n/n	n/n	n/+	n/n	n/n	n/n

n–FC absolute value < 2; ǀLog_2_FCǀ<1

+–Log_2_FC > 1

-–Log_2_FC < -1

*—p-value < 0.05

### Analysis of differential expression in male samples

The analysis of differentially expressed genes of males revealed further changes (Fig 3A1, 3B1, 3C1 and 3D1). The genes *CG42751* (more than 84 times down), *spn-B* (more than 8.6 times down) and the genes *mei-9* (2 times up), *mei-41* (2.6 times up), *mus309* (2 times up), *Cyp4e2* (more than 2.2 up) are differentially expressed 48 and 72 hours after the exposure respectively. This effect was observed only after 5 cGy, 10 cGy and 20 cGy dose irradiation. Such extended expression changes may reflect the fact that these genes are genes of late response to stress. For example, the expression of the gene *mei-9* encoding the protein of nucleotide excision repair and DNA mismatch repair is shown to be activated in response to UV radiation 12 hours after impact and later [101]. Overexpression of gene *Cyp4e2* and down-regulation of gene *CG42751* revealed in this study are matched with results of analysis of response to different stressors by *Drosophila melanogaster* transcriptome sequencing [23]. Although the function of the gene *CG42751* is still unknown, its expression changes were identified in response to oxidative stress [102], and it is known that indirect effects of the ionizing radiation are mediated by the induction of free radicals [103]. Gene *Cyp4e2* of the cytochrome P450 gene family plays a role in the regulation of circadian rhythms [104] and in response to different stresses, mostly chemical stressors. For example, overexpression of this gene is identified in different stress-resistant *Drosophila melanogaster* strains [105]. The genes *mei-9* and *mei-41* regulate DNA repair in somatic cells [106, 107], moreover, gene *mei-41* is required for hormesis, since lack of the hormetic effect was shown in mutants with inactive *mei-41* [14]. In our experiments, this gene is overexpressed (2.6 times) in response to 10 cGy dose radiation. The overexpression of the gene *spn-B* participating in the double-strand break DNA repair is not necessary for increase of the lifespan in response to low dose irradiation (30 cGy) [108], and it is downregulated 8.6–39 times after 48 hours in response to 5 cGy, 10 cGy and 20 cGy irradiation.

Gene Cyp6a20, encoding protein cytochrome P450 6a20, which plays a role in immune response and regulating fly behavior [78], is overexpressed immediately after impact of 5 cGy irradiation (2.3 times) and down-regulated in response to 40 cGy irradiation (2.5 times). Probably, effects of genes of rapid reaction to radiation differ among samples exposed to different doses of radiation because at the moment of measurement of higher cumulative radiation doses these gene expressions are already inversely compared with lower cumulative radiation doses, and consequently, shorter exposure time.

Similar regularity of gene *CG18180* is observed in male samples. This gene is overexpressed immediately after 5 and 10 cGy exposure, but there are no expression changes in response to 20, 40 cGy irradiation. The difference between the time of the start of exposure and the measurement may also explain the mismatch between these results and the results of gene expression analysis by RNA-Seq, which identified down regulation of *Cyp6a20* and *CG18180* genes [23]. The gene *PCNA*, participating in DNA repair (nucleotide-excision repair, mismatch repair) [109] and DNA replication [65], is overexpressed in response to 20 cGy irradiation 24 hours (2.1 times) and 48 hours (2.8 times) after the impact. This result may characterize this gene as a gene of long-term radiation stress response. The expression of gene *Hsp70Aa* is upregulated immediately after 20 cGy radiation exposure (2 times) and then 72 hours (4.8 times) after the impact.

### Analysis of differential expression in female samples

The analysis of differentially expressed genes in female samples in response to low dose radiation exposure did not reveal any clear effects (S1 File). Most of them have high standard deviation and are very low, although they are higher than the biological variability.

The gene *mus309* responsible for DNA damage signaling and DNA repair [74] is overexpressed (3.7 times) immediately after the 5 cGy dose irradiation. The gene *CG13323* with unknown function is overexpressed in response to radiation exposure at dose 10 cGy (2.8 times) and 40 cGy (2.2 times) 6 hours after impact. This fact may reflect the participation of the *CG13323* gene in radiation response. The gene *CG6295*, which plays a role in lipid metabolism, [89] is highly overexpressed (more than 15000 times) in response to 10 cGy irradiation 6 hours after exposure. The radiation induced production of ROS and RNS is known to lead to lipid metabolism disturbance [110]. But the expression of the *CG6295* gene was down regulated in response to 20 cGy irradiation in other research [23]. Such a mismatch may be explained by the difference in time of exposure and analysis. The low expression level (22.6 times down) of the gene *Brca2* participating in DNA repair [68] is observed after the 20 cGy exposure after 48 hours, but standard deviation is high. The gene *Hsp70Aa* is down-regulated (21 times) in response to 10 cGy irradiation and overexpressed (3.2 times up) in response to 40 cGy irradiation after 6 hours and 48 hours respectively. The expression changes of this gene involved in heat shock response [97] in both males and females after radiation exposure may confirm the existence of a non-specific stress response mechanism. It is interesting to mention that gene *CG18180* is overexpressed in response to 5 cGy irradiation after 24 hours (2.5 times) and to 10 cGy irradiation 6 and 24 (more than 2.6 times up) hours after exposure respectively in females, whereas it is overexpressed just after exposure in the male samples. Although the function of gene *CG18180* is still unknown, there is an assumption that this gene participates in immune response [83] and in response to different types of stresses [84, 85]. Perhaps the differences in the dynamics of *CG18180* gene expression are responsible for sex-specific changes of lifespan of wild-type *Drosophila melanogaster* individuals after low dose radiation exposure. Gene *GstE3*, the glutathione S‐transferase playing a role in detoxification phase II [81], is overexpressed more than 20 times in response to 40 cGy irradiation after 24 hours, but in other research [23], the downregulation of this gene expression after 20 cGy radiation impact in males and females was observed, which may be explained by the difference in analysis time after exposure.

Sex-specific responses to different stimulus have been confirmed by many experiments. Also, hormesis effects of the same stresses depend on sex [111]. This difference may be explained by the fact that the same genes in individuals of different sexes have to act in various environments, although their functions are identical. For example, increased sexual activity reduces immunity in males [112]. Such a specific immune response regulation may be revealed also under other conditions, and sex-specific expression changes of *CG18180* may be the consequence. Also, expression of this gene was shown to change in response to starvation and cold impact [85].

To sum up, we revealed that expression profiles of the 29 genes under research 72 hours after low dose irradiation from 5 cGy to 40 cGy are different in males and females of *Drosophila melanogaster* wild-type strain *Canton-S*. The gene *Clk*, responsible for circadian rhythm regulation, is not differentially expressed under experiment conditions, although previously, expression changes of the gene of this pathway in response to 20 cGy dose irradiation by enrichment analysis in 5-day-old flies were shown [23]. The genes s*pn-B*, *mei-9*, *mei-41*, *mus309* participating in the DNA repair and the response to different stresses are overexpressed in males 48 and 78 hours after radiation exposure, which may confirm their late transcriptional activation in response to radiation stress, and probably plays key role in extension of lifespan after the exposure to low doses of γ-irradiation. The expression of the gene *mus309* is changed in both males and females, but the expression profiles are different: this gene, after 72 hours, is overexpressed in males more than twofold in response to 20 cGy irradiation and in females fourfold immediately after exposure of 5 cGy radiation impact. Reduced expression of the gene *CG42751* with unknown function may be evidence of its role in changed lifespan and in the stress-response reaction to radiation. The expression changes of the gene *Hsp70Aa* (overexpressed more than threefold in response to 40 and 20 cGy after 6, 48 hours in females, and 24, 72 hours in males respectively, and down-regulated six hours after 10 cGy irradiation in females by 20 times) involved in heat shock response [97] in both males and females after radiation exposure may confirm the existence of a non-specific stress response mechanism. The dynamics of the expression change of gene *CG18180*, playing a role in immune response, differs in males and females (overexpressed immediately in males and after 6–24 hours in females after 5 and 10 cGy dose irradiation), which may play a role in reducing median and maximal lifespan of females after this impact. The differences in gene expression profile reflect a sex-specific stress response and lifespan features in *Drosophila melanogaster* wild strain *Canton-S*.

## Conclusions

Although there were changes in various indicators of life expectancy after exposure to 5, 10, 20 and 40 cGy, according to our analyses, they were not caused by the changes of organism physiological functions in the *Drosophila melanogaster* individuals after treatment, and furthermore there were not dose-dependent changes in the expression profile of stress-response genes chosen for the present study. It also should be noted that the cases of low dose irradiation expression changes are characterized by high biological variability, displaying a stochastic nature of low dose radiation effects. These results demonstrate the nonlinear character of low dose radiation effects on the *Drosophila melanogaster* imago and reveal a possible role of the gene *CG18180* in sex-specific stress response and lifespan features.

## Supporting Information

S1 File The mean relative gene expression with the standard deviation for three biological replicates after the radiation exposure in dose. Results for 5 cGy irradiation of males (Figure A1). Results for 10 cGy irradiation of males (Figure B1). Results for 20 cGy irradiation of males (Figure C1). Results for 40 cGy irradiation of males (Figure D1). Results for 5 cGy irradiation of females (Figure A2). Results for 10 cGy irradiation of females (Figure B2). Results for 20 cGy irradiation of females (Figure C2). Results for 40 cGy irradiation of females (Figure D2).
(DOC)

S1 Table Results of running the log rank and Gehan-Breslow-Wilcoxon tests. (DOCX)

S2 Table Primers for qPCR gene expression analysis. (XLS)

S3 Table Ct values from qPCR for three biological replicates after the radiation exposure. (XLSX)
