# Supplementary material for: Effect of Low Doses (5-40 cGy) of Gamma-irradiation on Lifespan and Stress-related Genes Expression Profile in Drosophila melanogaster
Source: PLoS One. 2015 Aug 6;10(8):e0133840. doi: 10.1371/journal.pone.0133840 (PMC4527671; doi:10.1371/journal.pone.0133840)
Supplement: S1 File — Results for 5 cGy irradiation of males (Figure A1). Results for 10 cGy irradiation of males (Figure B1). Results for 20 cGy irradiation of males (Figure C1). Results for 40 cGy irradiation of males (Figure D1). Results for 5 cGy irradiation of females (Figure A2). Results for 10 cGy irradiation of females (Figure B2). Results for 20 cGy irradiation of females (Figure C2). Results for 40 cGy irradiation of females (Figure D2). (DOC) [file pone.0133840.s001.doc]

The mean relative gene expression with the standard deviation for three biological replicates after the radiation exposure in dose: A – 5 cGy, B – 10 cGy, C – 20 cGy, D – 40 cGy; 1 – males, 2 – females

**A1**

**B1**

**C1**

**D1**

**A2**

**B2**

**C2**

**D2**
