# Supplementary material for: Effect of Low Doses (5-40 cGy) of Gamma-irradiation on Lifespan and Stress-related Genes Expression Profile in Drosophila melanogaster
Source: PLoS One. 2015 Aug 6;10(8):e0133840. doi: 10.1371/journal.pone.0133840 (PMC4527671; doi:10.1371/journal.pone.0133840)
Supplement: S1 Table — (DOCX) [file pone.0133840.s002.docx]

**Results of running the log rank test and Gehan-Breslow-Wilcoxon test for males**

**===== log-rank test (Mantel-Cox test, Mantel-Haenszel test) =====**

N Observed Expected (O-E)^2/E (O-E)^2/V

Control 1044 1044 994 2.5020 4.4490

5 cGy 423 423 418 0.0597 0.0799

10 cGy 426 426 494 9.4835 13.3343

20 cGy 391 391 355 3.5696 4.5667

40 cGy 438 438 460 1.0529 1.4651

Chisq= 19.1 on 4 degrees of freedom, p = 0.000739

The cohorts are significantly different (p < 0.001)

**===== pairwise log-rank tests (Mantel-Cox test, Mantel-Haenszel test) =====**

N Observed Expected (O-E)^2/E (O-E)^2/V

Control 1044 1044 1032 0.145 0.561

5 cGy 423 423 435 0.343 0.561

Chisq= 0.6 on 1 degrees of freedom, p = 0.454

No statistical significant difference (p > 0.05) between these cohorts could be detected

N Observed Expected (O-E)^2/E (O-E)^2/V

Control 1044 1044 979 4.25 14.9

10 cGy 426 426 491 8.49 14.9

Chisq= 14.9 on 1 degrees of freedom, p = 0.000114

These cohorts are significantly different (p < 0.001)

N Observed Expected (O-E)^2/E (O-E)^2/V

Control 1044 1044 1053 0.0691 0.296

20 cGy 391 391 382 0.1903 0.296

Chisq= 0.3 on 1 degrees of freedom, p = 0.586

No statistical significant difference (p > 0.05) between these cohorts could be detected

N Observed Expected (O-E)^2/E (O-E)^2/V

Control 1044 1044 1016 0.78 2.89

40 cGy 438 438 466 1.70 2.89

Chisq= 2.9 on 1 degrees of freedom, p = 0.0889

No statistical significant difference (p > 0.05) between these cohorts could be detected

**===== Generalized Wilcoxon test =====**

N Observed Expected (O-E)^2/E (O-E)^2/V

Control 1044 543 534 0.135 0.337

5 cGy 423 221 214 0.236 0.432

10 cGy 426 203 236 4.729 9.004

20 cGy 391 207 195 0.780 1.393

40 cGy 438 228 223 0.135 0.249

Chisq= 9.5 on 4 degrees of freedom, p = 0.0495

The cohorts are significantly different (p < 0.05)

**===== pairwise generalized Wilcoxon tests =====**

N Observed Expected (O-E)^2/E (O-E)^2/V

Control 1044 538 541 0.0130 0.0709

5 cGy 423 220 217 0.0324 0.0709

Chisq= 0.1 on 1 degrees of freedom, p = 0.79

No statistical significant difference (p > 0.05) between these cohorts could be detected

N Observed Expected (O-E)^2/E (O-E)^2/V

Control 1044 553 526 1.42 7.28

10 cGy 426 206 234 3.19 7.28

Chisq= 7.3 on 1 degrees of freedom, p = 0.00697

These cohorts are significantly different (p < 0.01)

N Observed Expected (O-E)^2/E (O-E)^2/V

Control 1044 535 543 0.103 0.601

20 cGy 391 206 199 0.281 0.601

Chisq= 0.6 on 1 degrees of freedom, p = 0.438

No statistical significant difference (p > 0.05) between these cohorts could be detected

N Observed Expected (O-E)^2/E (O-E)^2/V

Control 1044 539 540 0.00471 0.025

40 cGy 438 227 225 0.01128 0.025

Chisq= 0 on 1 degrees of freedom, p = 0.874

No statistical significant difference (p > 0.05) between these cohorts could be detected

**Results of running the log rank test Gehan-Breslow-Wilcoxon test for females**

===== log-rank test (Mantel-Cox test, Mantel-Haenszel test) =====

N Observed Expected (O-E)^2/E (O-E)^2/V

Control 1017 1017 1010 0.0551 0.101

5cGy 381 381 404 1.3267 1.755

10cGy 318 318 246 20.9700 25.502

20cGy 457 457 302 79.1573 98.796

40cGy 438 438 649 68.5131 108.629

Chisq= 199 on 4 degrees of freedom, p < 2.2e-16

The cohorts are significantly different (p < 0.0001)

**===== pairwise log-rank tests (Mantel-Cox test, Mantel-Haenszel test) =====**

N Observed Expected (O-E)^2/E (O-E)^2/V

Control 1017 1017 997 0.39 1.53

5cGy 381 381 401 0.97 1.53

Chisq= 1.5 on 1 degrees of freedom, p = 0.216

No statistical significant difference (p > 0.05) between these cohorts could be detected

N Observed Expected (O-E)^2/E (O-E)^2/V

Control 1017 1017 1072 2.81 15.8

10cGy 318 318 263 11.44 15.8

Chisq= 15.8 on 1 degrees of freedom, p = 6.97e-05

These cohorts are significantly different (p < 0.0001)

N Observed Expected (O-E)^2/E (O-E)^2/V

Control 1017 1017 1132 11.7 56.7

20cGy 457 457 342 38.7 56.7

Chisq= 56.7 on 1 degrees of freedom, p = 5.03e-14

These cohorts are significantly different (p < 0.0001)

N Observed Expected (O-E)^2/E (O-E)^2/V

Control 1017 1017 886 19.4 57.6

40cGy 438 438 569 30.2 57.6

Chisq= 57.6 on 1 degrees of freedom, p = 3.29e-14

These cohorts are significantly different (p < 0.0001)

**===== Generalized Wilcoxon test =====**

N Observed Expected (O-E)^2/E (O-E)^2/V

Control 1017 526 521 0.0434 0.109

5cGy 381 182 212 4.2965 8.044

10cGy 318 185 142 12.9409 21.681

20cGy 457 277 194 35.6342 60.078

40cGy 438 176 276 36.6338 76.198

Chisq= 139 on 4 degrees of freedom, p < 2.2e-16

The cohorts are significantly different (p < 0.0001)

**===== pairwise generalized Wilcoxon tests =====**

N Observed Expected (O-E)^2/E (O-E)^2/V

Control 1017 536 512 1.13 6.08

5cGy 381 185 209 2.75 6.08

Chisq= 6.1 on 1 degrees of freedom, p = 0.0136

These cohorts are significantly different (p < 0.05)

N Observed Expected (O-E)^2/E (O-E)^2/V

Control 1017 507 539 1.88 13.3

10cGy 318 180 148 6.88 13.3

Chisq= 13.3 on 1 degrees of freedom, p = 0.000267

These cohorts are significantly different (p < 0.001)

N Observed Expected (O-E)^2/E (O-E)^2/V

Control 1017 495 552 5.85 32.1

20cGy 457 264 207 15.58 32.1

Chisq= 32.1 on 1 degrees of freedom, p = 1.47e-08

These cohorts are significantly different (p < 0.0001)

N Observed Expected (O-E)^2/E (O-E)^2/V

Control 1017 560 487 10.7 49

40cGy 438 190 262 19.9 49

Chisq= 49 on 1 degrees of freedom, p = 2.56e-12

These cohorts are significantly different (p < 0.0001)
